# Targeting TIGIT for Immunotherapy of Cancer: Update on Clinical Development

**DOI:** 10.3390/biomedicines9091277

**Published:** 2021-09-21

**Authors:** Anand Rotte, Srikumar Sahasranaman, Nageshwar Budha

**Affiliations:** 1 Arcellx, Gaithersburg, MD 20878, USA; 2 Doloxe, Santa Clara, CA 95050, USA; 3 BeiGene USA, Inc, San Mateo, CA 94608, USA; sri.sahasranaman@beigene.com (S.S.); nageshwar.budha@beigene.com (N.B.)

**Keywords:** TIGIT, immune checkpoints, immunotherapy and cancer

## Abstract

Immune checkpoint blockers have dramatically improved the chances of survival in patients with metastatic cancer, but only a subset of the patients respond to treatment. Search for novel targets that can improve the responder rates and overcome the limitations of adverse events commonly seen with combination therapies, like PD-1 plus CTLA-4 blockade and PD-1/PD-L1 plus chemotherapy, led to the development of monoclonal antibodies blocking T-cell immunoglobulin and ITIM domain (TIGIT), a inhibitory checkpoint receptor expressed on activated T cells and NK cells. The strategy showed potential in pre-clinical and early clinical studies, and 5 molecules are now in advanced stages of evaluation (phase II and above). This review aims to provide an overview of clinical development of anti-TIGIT antibodies and describes the factors considered and thought process during early clinical development. Critical aspects that can decide the fate of clinical programs, such as origin of the antibody, Ig isotype, FCγR binding, and the dose as well as dosing schedule, are discussed along with the summary of available efficacy and safety data from clinical studies and the challenges in the development of anti-TIGIT antibodies, such as identifying patients who can benefit from therapy and getting payer coverage.

## 1. Introduction

The idea of using immune response against abnormal cells in the body to treat cancer has been tested in the past few decades and evolved from using recombinant cytokines to adoptive cell transfer [1,2]. The first generation of immunotherapies like high-dose interleukin-2 were limited by low response rates and high incidence of serious adverse events, but the durability of response encouraged further research in the field [3,4,5]. Discovery of checkpoints of T-cell activation and development of monoclonal antibodies targeting the checkpoints dramatically changed the outcomes of immunotherapy [6,7,8,9,10,11,12]. Cytotoxic T-lymphocyte-associated protein 4 (CTLA-4) and programmed cell death protein 1 (PD-1) were the early targets that were discovered and characterized in the late 1980s and early 1990s, respectively [13,14,15,16,17,18,19]. Both CTLA-4 and PD-1 have been shown to be reliable targets, and to date, seven drugs have been approved for different types of cancers, such as melanoma and lung cancer [20,21,22,23,24]. In addition to monotherapy, combination of CTLA-4 and PD-1 blockers is also approved for treatment of multiple cancer types [23]. While the CTLA-4 and PD-1 blockers had decent and durable response rates, a large fraction of patients did not respond to the treatment, and the incidence of serious adverse events was high in the responding patients [25,26,27]. The need for safer targets that can be blocked or activated to achieve reasonable anti-tumor response with manageable adverse events and that can be combined with PD-1/PD-L1 blockers or other immune checkpoint blockers led to the identification of T-cell immunoglobulin and ITIM domain (TIGIT), an inhibitory immune checkpoint, and the development of anti-TIGIT antibodies.

TIGIT is considered as an important target mainly because of its expression profile (natural killer cells (NK cells), cytotoxic CD8^+^ T cells and regulatory T cells (Tregs) [28]. More importantly, the phenotype of *Tigit^−/−^* mouse was reported to be mild, and the knockout mice did not spontaneously develop autoimmunity, indicating a comparatively milder safety profile [29]. Multiple review articles have discussed the significance, biology, signaling, and role in immune response of TIGIT alone or along with other recently identified immune checkpoints, such as T-cell immunoglobulin-3 (TIM-3) and lymphocyte activation gene 3 (LAG-3) [28,30,31,32,33,34]. However, the critical aspects of TIGIT and anti-TIGIT antibodies that are relevant for the early clinical development, such as origin of antibody (humanized or fully human), immunoglobulin G (IgG) backbone, and Fcγ receptors (FcγRs), and factors considered in determining the dose are not discussed in detail in previous reviews. Dose, regimen, and other considerations have significant impact on the efficacy and safety of the lead molecule and can thereby impact the success or failure of a clinical program. Therefore, the current review was undertaken to provide readers a source of information on the points considered during early clinical development of monoclonal antibodies targeting TIGIT and provide an up-to-date summary of efficacy and safety findings. To give the reader a complete idea of TIGIT, biology of the receptor and its role in immune response are briefly discussed in this review along with the aspects of clinical development.

## 2. Tigit

### 2.1. Discovery

TIGIT was reported by scientists from Genentech and Washington University independently in 2008 through a genomic search for T-cell-specific genes that encode potential inhibitory receptors and as a novel immunoreceptor on human follicular B helper T cells (TFH) that interacted with follicular DCs via polio virus receptor (PVR), respectively [35,36]. TIGIT gene, located on chromosome 3q13.31, encodes a 244-amino acid protein consisting of single extracellular immunoglobulin domain, a type 1 transmembrane region, and a single intracellular ITIM domain [35]. TIGIT receptor belongs to the nectin and nectin-like receptors superfamily [32].

### 2.2. Expression

TIGIT expression is mainly seen on resting CD4^+^CD25^hi^ Treg cells, activated T cells, NK cells, NKT cells, and memory T cells (Table 1). Naïve CD4^+^ T cells do not express TIGIT, but its expression is induced at mRNA levels upon activation [35]. TIGIT has been reported as marker for CD8+ T-cell exhaustion and is also a characteristic marker for Tregs in the tumor microenvironment [7,28,29,37,38].

### 2.3. Ligands and Cells Expressing Ligands

TIGIT is believed to act by competing with T-cell costimulatory receptors, CD226 (also known as DNAX accessory molecule-1, DNAM1) and CD96 for CD155 (also known as polio virus receptors, PVR and nectin like protein-5), CD112 (also known as PVR-related 2, PVRL-2 and nectin-2) and CD113 (also known as PVRL-3 and nectin 3). The main ligand for TIGIT is CD155, and its binding affinity with CD112 and CD113 was reported to be lower compared to CD155 [32]. Recently, a novel ligand for TIGIT, nectin-4, was identified. The binding affinity of nectin-4 was comparable to CD155, and it was concluded to be the only member of nectin-family proteins that interacted exclusively with TIGIT and not with CD226, CD96, or with CD112 [39].

CD155 expression is mainly reported on DCs, T cells, B cells, and macrophages, whereas CD112 is widely expressed on both hematopoietic and non-haematopoietic tissues, including bone marrow, lung, pancreas, and kidney [40,41]. CD113 expression is limited to non-hematopoietic tissues, such as lung, liver, testis, kidney, and placenta [42]. Several human cancers are reported to overexpress CD155 and CD112 [43,44,45]. Interestingly, interferon-γ (IFN- γ) was shown to up-regulate the expression of CD155 on human vascular endothelial cells, a mechanism similar to induction of PD-1/PD-L1 pathway [46].

### 2.4. Regulation of Immune Response

**Figure 1:**
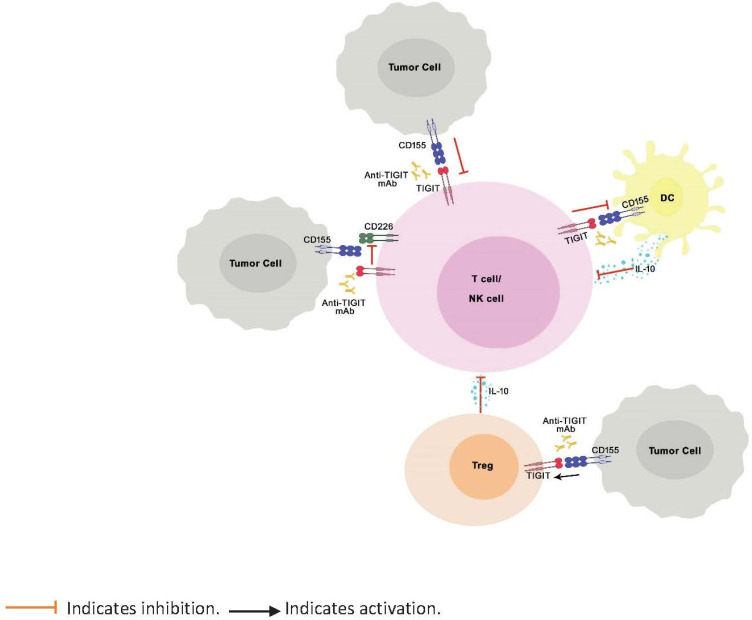
Role of TIGIT in regulation of immune response. TIGIT competitively inhibits binding of CD226 to CD155 and impairs the CD226-mediated activation of T cells and NK cells. Binding of TIGIT to its ligand CD155 results in activation of inhibitory signals in T cells and NK cells. TIGIT binding to CD155 on APCs results in IL-10 production, decreased IL-12 production (not shown in the illustration), and indirect inhibition of T cells. Finally, TIGIT signaling enhances the immunosuppressive functions of Tregs.

TIGIT is a negative regulator of immune response known to bind to PVR ligands with greater affinity and outcompete the costimulatory receptors, CD226 and CD96, expressed on T cells, thereby inhibiting the activation, proliferation, and differentiation of T cells (Figure 1). Further, TIGIT engagement ensures the survival of inhibited T cells by activating cell survival pathways. [7]. TIGIT activation on NK cells was shown to inhibit cytotoxic granule polarization and IFN-γ production and decrease NK cell cytotoxicity [30,47]. In addition, TIGIT interaction on Tregs skews the cytokine balance, suppresses Th1 or Th17 phenotype, and induces Th2 phenotype [29,48]. However, unlike CTLA-4 and PD-1, which, when knocked out in mice, are known to manifest as severe and spontaneous autoimmune phenotype [17,49,50,51], TIGIT knock-out mice do not spontaneously develop autoimmune phenotype, indicating mild to moderate control of TIGIT over immune response [29].

### 2.5. Target for Cancer Immunotherapy

Even before the discovery of TIGIT, its ligands were known to be upregulated on the surface of tumor cell surface. Expression of nectin family of proteins and their role in cell adhesion and survival was reported in tumors from epithelial origin, such as non-small cell lung cancer, colon cancer, and metastatic neuroblastoma, and also tumors from hematopoietic origin, such as myeloid leukemia [43,52,53,54,55]. High expression of CD155 was shown to be an independent prognostic marker and predictor of poor clinical outcome in breast cancer patients [56]. The recently discovered ligand for TIGIT, nectin 4, was shown to be overexpressed in breast, bladder, lung, and pancreatic cancers [57]. On the other hand, TIGIT expression was also reported to be upregulated on lymphocytes in tumor microenvironment. Studies showed TIGIT expression on CD8^+^, CD4^+^ T cells, and NK cells paralleled to that of PD-1 in hepatocellular, lung, and colorectal cancers and in Hodgkin’s lymphoma [58,59,60,61,62,63,64,65].

The potential of targeting TIGIT was shown using in-vivo mouse models for cancers and chronic viral infection (Table 2). Researchers showed that blockade of TIGIT along with PD-L1 enhanced CD8+ T-cell effector function and tumor and viral clearance [37,66,67]. Studies in tumor-bearing *Tigit^+/+^* and *Tigit^−/−^* mice demonstrated increased TIGIT expression on tumor infiltrating lymphocytes and lack of TIGIT in Tregs to be critical for immune suppression in tumor microenvironment [29,68]. Administration of monoclonal antibodies against TIGIT was shown to increase survival rate in mouse models for ovarian cancer. Study found that the anti-TIGIT antibody treatment reduced CD4+ Tregs but did not affect the proportion of CD4+ T-helper cells, CD8+ T cells, or NK cells [69].

## 3. Anti-Tigit Antibodies in Development

Targeting TIGIT-PVR pathway has gained importance in the recent months, and several biotech/pharmaceutical companies are working on development of anti-TIGIT antibodies. As of June 2020, 15 antibodies targeting TIGIT-PVR pathway are being commercially developed and are in various stages of clinical development. The list of molecules, details of antibody isotypes, Fc status, and current status is presented in Table 3. Nine molecules are in clinical trials, and tiragolumab, developed by Genentech and ociperlimab, developed by BeiGene, are in the most advanced stage of development (Phase III). In January 2021, FDA granted breakthrough therapy designation, a pathway designed to accelerate the development and review of data for tiragolumab plus atezolizumab combination as first-line treatment of people with metastatic non-small cell lung cancer (NSCLC) whose tumors have high PD-L1 expression with no EGFR or ALK genomic tumor aberrations [70]. Nearly half of the antibodies developed have IgG1 back bone, whereas the antibody developed by Astellas Pharma has IgG4 back bone (Table 3).

In addition to monospecific antibodies, researchers are also developing bispecific antibody that co-targets PD-L1 and TIGIT. Generation and characterization of a multivalent bispecific antibody consisting of tetravalent anti-PD-L1 Fc-fusion nanobody and tetravalent anti-TIGIT nanobody was recently reported [71]. The bispecific antibody showed high specificity and affinity to primate PD-L1 and TIGIT and had significantly higher anti-tumor activity compared to PD-L1 antibody in mouse models. Benefits of bispecific antibodies targeting multiple immune checkpoints need to be demonstrated in clinical studies.

In the following sections, factors considered during the early clinical development of antibodies are discussed in relation to the development of anti-TIGIT antibodies.

### 3.1. Factors Considered during Development

Several factors, such as origin of the antibody backbone (mouse, chimeric, humanized, or fully human), IgG backbone for the antibody, FcγR binding status, and dose, play a key role in the development and eventual clinical success of the antibody. Details of the factors are discussed in the following sections.

### 3.2. Origin

The origin of antibodies intended for therapeutic application can significantly impact the clinical success of the molecules. Antibodies generated in mice were shown to induce production of anti-mouse antibodies in patients, which increased the clearance of antibody-based drugs. Chimeric antibodies, which have part of their protein structure from human origin and the other part from animal origin, were expected to be better but still suffered due to anti-drug antibodies. Humanized antibodies, which have protein sequences that closely matched to that of humans and fully human antibodies that do not have any protein sequence from mouse (or other animal) origin, are expected to be least immunogenic and have very low chances of anti-drug antibody development [72,73]. All the approved immune checkpoint blockers to date are either humanized (pembrolizumab, atezolizumab) or fully human (ipilimumab, nivolumab), and the majority of the anti-TIGIT antibodies in clinical development are fully human antibodies (Table 3).

### 3.3. IgG Isotype and FcγR Binding

Almost all the commercially developed immune checkpoint blocking antibodies have IgG backbone. IgG based antibodies are known to interact with FcγR on innate effector immune cells through their Fc-region and induce antibody dependent cellular cytotoxicity (ADCC) in the target cells. ADCC is a non-phagocytic mechanism through which innate immune cells including macrophages, DCs, neutrophils, and NK cells, kill the antibody-bound target cells [7,74,75,76,77,78]. Activation of ADCC through the binding of Fc-γRI (CD64), Fc-γ RIIa, Fc-γ RIIc (CD32), and Fc-γ RIIIa (CD16) triggers the release of cytotoxic mediators, such as tumor necrosis factor-α (TNF-α), perforin, granzyme, and reactive oxygen species (ROS), from effector immune cells on to the target cell surface and result in lysis of target cells.

Affinity of the antibody to Fcγ receptors of effector cells is the key to induction of ADCC and is mainly dependent on the antibody backbone. IgG1 backbone has highest affinity to all the three stimulatory FcγRs and induces significant ADCC, whereas IgG2 backbone does not bind to FcγRs and does not induce ADCC [7]. ADCC is the most common factor that is considered during the development of therapeutic antibodies. Induction of ADCC is a desired effect for antibodies targeting receptors on cancer cells and has been shown to be a contributor to the anti-tumor activity of monoclonal antibodies [79,80,81].

While ADCC is considered beneficial for the antibody-drug conjugates, its contribution to the activity of immune checkpoint blockers is not completely clear. For example, PD-1-blocking antibodies pembrolizumab and nivolumab that showed promising success in the treatment of cancer have IgG4 backbone and are known to have relatively lower binding affinity to FcγRs. They also did not show significant ADCC activity in the in-vitro models [82]. Similarly, tislelizumab, an anti-PD-1 antibody with IgG4 backbone is specifically designed to minimize FcγRs [83,84,85]. However, the anti-CTLA-4 antibody, ipilimumab, which is IgG1 based and known to induce ADCC, has been successful compared to IgG2 based anti-CTLA-4 antibody, tremelimumab, which does not have ADCC activity.

The FcγR binding region of anti-TIGIT antibodies in clinical development is active in some of the molecules and inactivated in others. Based on publicly available information, six out of nine molecules, including tiragolumab, ociperlimab, vibostolimab, EOS-448, etigilimab, and AGEN-1307, have active FcγR binding region, whereas three molecules, including domvanalimab, BMS-986207, and CASC-674, have inactive FcγR binding region (Table 3). The FcγR binding region in AGEN-1307 is mutated to enhance the binding of the antibody with Fcγ receptors and increase its ADCC activation [86]. It remains to be seen if the presence or absence of FcγR binding region in the antibody would have an impact on the clinical efficacy of anti-TIGIT antibodies.

### 3.4. Dose

Various factors, including target binding affinity; pharmacodynamic factors, like saturation of downstream biomarker response and concentration at which optimal receptor occupancy is achieved; pharmacokinetic factors, like saturation of target-mediated elimination pathway and anti-drug antibodies that reduce target drug concentration, dose, or exposure-response relationships for efficacy and safety; and maximum tolerated dose and dose at which drug is expected to have maximum effect, are considered before selecting the dose for advanced studies. PK-PD models, simple mechanistic models, as well as complex mechanistic models, such as quantitative systems pharmacology models, are typically used to simulate the dose that achieves optimal target occupancy and achieves desired pharmacological effect. In cases where information is not completely available to develop PK-PD models or mechanistic models, available literature information from related molecules is used to propose the dose that can possibly have desired response.

Results from Phase I studies across multiple clinical programs demonstrate that anti-TIGIT antibodies are well tolerated (Table 4). Studies used different dose ranges of the antibody and used either every two weeks (Q2W) or every three weeks (Q3W) administration regimen. Dose-limiting toxicities were not recorded during monotherapy or in combination with anti-PD-1 antibody for any of the anti-TIGIT antibodies in clinical development, indicating molecules against this target have broad therapeutic index. Highest dose of anti-TIGIT antibody evaluated was 20 mg/kg Q2W for etigilimab (Table 4). Clinical activity (objective response rate) observed after anti-TIGIT antibody monotherapy was minimal to none, indicating combination therapy with anti-PD1 or PD-L1 or other agents is needed. Complete peripheral TIGIT receptor occupancy was observed for most drugs at very low doses. For example, tiragolumab evaluated doses starting at 2 mg, and complete receptor occupancy was observed at 30 mg dose. Similarly, ociperlimab evaluated doses starting at 50 mg, and complete receptor occupancy was observed at this dose and above. Domvanalimab reported complete receptor occupancy at the dose of 0.5 mg/kg [87].

Based on the concentration at which maximum receptor occupancy was achieved, and based on the concentration at which early clinical activity was noticed, a dose of 600 mg was proposed for tiragolumab phase II studies [88]. Tiragolumab’s recommended phase II dose (RP2D) of 600 mg Q3W is approximately 20-fold higher than the initial dose at which complete peripheral receptor occupancy was observed. Similarly, ociperlimab RP2D was 900 mg Q3W in published clinical trials, which is ~18-fold higher than the initial dose at which complete receptor occupancy was observed (NCT04746924). Vibostolimab appears to be investigating RP2D of 200 mg Q3W based on the clinical study posted (NCT04738487); however, no information-receptor on occupancy or other pharmacodynamic biomarker data are available. Though other molecules, including domvanalimab, BMS986207, and EOS-448, also entered into phase II trials (Table 3), dose of the antibody has not been publicly disclosed at the time of data compilation.

### 3.5. Safety

Anti-TIGIT antibodies were found to be generally well tolerated when administered as monotherapy as well as when administered in combination with PD-1/PD-L1 blockers (Table 4). Most common adverse events reported in more than 10% patients included fatigue and pruritus; both were Grade 1. Two Grade 2 events, anemia and diarrhea, were reported in two patients treated with vibostolimab monotherapy. There were no Grade 3–5 events reported with anti-TIGIT antibody monotherapy.

## 4. Clinical Status

List of ongoing clinical trials registered on clinicaltrials.gov (accessed on 25 August 2021) is presented in Table 5. Twenty-three clinical trials were found to be ongoing at the time of data compilation, with 22 trials actively recruiting patients. Tiragolumab is comparatively in advanced stages of development with two phase III trials and two phase II trials. Interim results from phase II randomized trial evaluating the benefits of combining tiragolumab with atezolizumab have been presented at Annual Meeting of AACR 2020 [88]. Study randomized locally advanced unresectable or metastatic PD-L1-selected non-small cell lung cancer (NSCLC) patients in a 1:1 ratio into placebo plus atezolizumab or tiragolumab plus atezolizumab groups. The dose and regimen of atezolizumab in both groups was 1200 mg administered via intravenous (IV) infusion every three weeks. In addition to atezolizumab, patients either received matched placebo or tiragolumab 600 mg, administered via IV infusion every three weeks. At the time of data-cut, objective response rate (ORR) was reported as 16% (95% confidence interval, 7–26) in the placebo group versus 31% (19–43) in the tiragolumab group (odds ratio, 2.6). Progression-free survival was reported as 3.6 months and 5.4 months, respectively. The incidence of adverse events was not different between the groups, and both cohorts had similar rate of serious adverse events (35% vs. 34%, respectively). Interestingly, response to combination treatment was reported to correlate with PD-L1 expression, and patients with ≥50% PD-L1 expression had an ORR of 66% and did not reach median PFS, whereas patients with lower PD-L1 expression had an ORR of 16% and a median PFS of 4.0 months [88].

Multiple phase III studies have also been initiated for ociperlimab and are currently enrolling patients (Table 5). Findings from phase I dose-escalation study were presented at Annual Meeting of ASCO 2021 [89]. The study aimed at evaluating safety and preliminary anti-tumor activity of ociperlimab in combination with tislelizumab and determine RP2D of the combination. Twenty-four patients with advanced solid tumors were enrolled in the study and were administered the combination. At the data-cut off (median follow-up time, 17 weeks), authors noted that there were no dose-limiting toxicities; one patient had partial response, and nine patients had stable disease. Authors also noted that ociperlimab exposure increased dose proportionally and sustained TIGIT receptor occupancy was seen at ≥50 mg doses.

Results from dose finding study of vibostolimab as monotherapy and in combination with pembrolizumab were also presented at Annual Meeting of ESMO 2020 [90]. Study enrolled anti–PD-1/PD-L1-refractory NSCLC patients into vibostolimab (200 or 210 mg) monotherapy arm or the combination arm with the primary objective of evaluating safety and tolerability of vibostolimab when given alone or in combination with pembrolizumab. Results showed that vibostolimab was well-tolerated, and the incidence of treatment-related adverse events (any grade) was similar between monotherapy and combination arms. ORR was 7% (2–20) patients in monotherapy group, and 5% (<1–18) in combination group. Treatment-related grade 3–4 adverse events were reported in 10 patients, and lipase increase and hypertension were the common events. One patient in the combination group died due to pneumonitis [90].

## 5. Challenges

Success of anti-TIGIT antibodies mainly depends on identifying the prognostic biomarkers of response and the patients who would respond to the treatment. In the last five years, multiple combinations of anti-PD-1/PD-L1 antibodies, including combination with ipilimumab (CTLA-4), chemotherapy, and bevacizumab (VEGF), have been approved for the treatment of different types of solid tumors. In addition, multiple CAR-T cell therapies, oncolytic viral therapies, and targeted therapeutics, such as BRAF inhibitors, MEK inhibitors, RTK inhibitors, and PARP inhibitors, have also been approved for the treatment of cancers. With several approved therapies available, it would be challenging to find the right subset of patients who could be benefited by anti-TIGIT monotherapy or combinations. Similarly, it would also be challenging to get market access and payer coverage anti-TIGIT antibodies because of available treatment options. As all the novel therapies including immunotherapy are priced high to cover the developmental expenses, insurance companies require to see reports of cost benefits before providing coverage. Multiple cost-effectiveness and cost-utility analyses are therefore needed to convince the payers to provide coverage.

## 6. Summary

To summarize, immunotherapy and checkpoint blockers have transformed the treatment landscape of cancer and improved the chances of survival dramatically, but there is an urgent need to increase the percentage of patients responding to treatment. Combination therapies, like PD-1 plus CTLA-4 blockade and PD-1/PD-L1 plus chemotherapy, have indeed increased the responder rates, but they are limited by increased incidence of serious, dose-limiting, adverse events, and a decent proportion of patients still do not respond to combination therapy. TIGIT can be a potential target for monotherapy as well as combination therapy with its promising efficacy and safety profile. Understandably, there is significant interest in the development of monoclonal antibodies targeting TIGIT receptors, and 15 pharmaceutical or biotech companies are currently pursuing clinical development of anti-TIGIT antibodies, with five molecules in advanced stages of clinical trials (phase II or above), including one molecule with breakthrough designation from U.S. FDA. Early clinical data have shown the importance of IgG1 isotype and active FcγR-mediated ADCC function in the activity of anti-TIGIT antibodies. Based on the data from maximum receptor occupancy and comparative literature evidence, a dose of 600 mg every two weeks was proposed for the advanced clinical studies for tiragolumab. While the current data from clinical studies assure safety and efficacy of anti-TIGIT antibodies, success of the molecules depends on patients utilizing the therapy. Further studies are needed to identify the biomarkers of response and the patient subset that are likely to respond to anti-TIGIT therapy and to evaluate combinations with chemotherapy and other blockers of checkpoints, such as LAG-3 and TIM-3. More importantly, almost all of the available clinical data on anti-TIGIT antibodies is from lung cancer patients, and the majority of ongoing clinical studies are also in lung cancer patients. Data from other types of cancers with high incidence, such as prostate; breast; colorectal; urinary/bladder and skin cancers (melanoma); hematological cancers, such as non-Hodgkin lymphoma; and brain cancers, such as glioblastoma, are needed for anti-TIGIT antibodies. The clinical utility of these antibodies is yet to be proven in these tumor types. Finally, further studies are also needed to demonstrate the cost advantages of anti-TIGIT antibody combinations to help the payers in making informed decisions on providing coverage and patient access to the treatment.

## Figures and Tables

**Table 1:** Cells expressing TIGIT receptors and ligands.

Receptor/Ligand	Cells/Tissues
TIGIT	Resting CD4^+^CD25^hi^ Treg cells, activated T cells, NK cells, NKT cells, memory T cells, and exhausted T cells
CD155 (PVR, nectin like protein-5)	DCs, T cells, B cells, macrophages, and cancer cells. Human vascular endothelial cells in response to IFN-γ
CD112 (PVRL-2, nectin-2)	Bone marrow, lung, pancreas, kidney, and some types of cancer
CD113 (PVRL-3, nectin-3	Lung, liver, testis, kidney, placenta, and some types of cancer
Nectin-4	Squamous epithelia, placenta, and some types of cancers

**Table 2:** Summary of key preclinical findings.

Study (First Author et al. [Reference])	Finding
Yu et al. 2009 [35] Boles et al. 2008 [36]	Discovery of TIGIT TIGIT is expressed on activated T cells, NK cells
Joller et al. 2014 [48]	TIGIT is expressed on distinct subset of Tregs that specifically suppress Th1 and Th17 cells
Kurtulus et al. 2015 [29]	TIGIT is a marker for CD8+ T-cell exhaustion *Tigit^−/−^* mice bearing colon cancer (MC38) or melanoma (B16F10) have significantly lower tumor growth
Johnston et al. 2014 [37]	TIGIT expression correlates with PD-1 in human cancer Co-blockade of TIGIT and PD-1 resulted in synergistic CD8^+^-mediated rejection of tumors
Chew et al. 2016 [38]	TIGIT is a marker for T-cell exhaustion
Zhang et al. 2018 [47]	TIGIT is associated with NK cell exhaustion TIGIT blockade prevented NK cell exhaustion and resulted in NK cell–dependent tumor immunity
Guillerey et al. 2018 [68]	Multiple myeloma progression is associated with high TIGIT expression on CD8+ T cells *Tigit^−/−^* mice bearing myeloma tumors (Vk12653) have lower tumor growth and longer survival TIGIT blockers suppressed multiple myeloma growth in mice
Hung et al. 2018 [67]	TIGIT expression is higher in CD8+ T cells and Tregs in brain of glioblastoma tumor (GL261)-bearing mice Co-blockade of TIGIT and PD-1 improved survival in glioblastoma tumor-bearing mice

**Table 3:** List of anti-TIGIT molecules in clinical development.

Generic Name	Type	FcγR Status	Company	Status
Tiragolumab (MTIG7192A)	Fully human IgG1	Active	Genentech	Phase III
Ociperlimab (BGB-A1217)	Humanized IgG1	Active	BeiGene USA, Inc	Phase III
Vibostolimab (MK-7684)	Fully human IgG1	Active	Merck & Co Inc	Phase II
Domvanalimab (AB-154)	Fully human IgG1	Inactive	Arcus Biosciences Inc	Phase II
BMS-986207	Fully human IgG1	Inactive	Bristol-Myers Squibb Co	Phase II
EOS-448	Fully human IgG1	Active	iTeos Therapeutics SA	Phase II
ASP-8374	Fully human IgG4	Inactive	Astellas Pharma Inc	Phase I
COM-902	Mouse/cyno cross-reactive fully human IgG1 antibody	NA	Compugen Ltd.	Phase I
Etigilimab	Fully human IgG1	Active	Mereo Biopharma Group Plc	Phase I
IBI-939	NA	NA	Innovent Biologics Inc	IND Filed
AGEN-1307	Fully human IgG1	Active and enhanced	Agenus Inc	Preclinical
CASC-674	Fully human IgG2a	Inactive	Seattle Genetics Inc	Preclinical
Anti-PVR Antibody (NB-6253)	NA	NA	Northern Biologics Inc	Preclinical
PH-804	NA	NA	Phio Pharmaceuticals Corp	Preclinical
TIGIT-PD-L1 dual	NA	NA	Aurigene Discovery Technologies Ltd.	Preclinical

NA, details not available.

**Table 4:** Studies reporting anti-TIGIT antibody dose and tolerability.

Drug	Phase	Dose and Regimen	Comment	Reference
Tiragolumab	Phase III Multiple Solid tumors	2 mg to 1200 mg Q3W RP2D: 600 mg Q3W	100% receptor occupancy seen at ≥30 mg and clinical activity observed at doses 400 mg to 600 mg. 600 mg Q3W was proposed as dose for Ph2 study.	Bendell et al. AACR 2020
Ociperlimab	Phase III	50 mg to 900 mg RP2D: 900 mg Q3W	100% receptor occupancy was observed at 50 mg, and linear PK was observed through 900 mg.	NCT04746924
Domvanalimab (AB-154)	Phase I NSCLC	0.5 mg/kg; 1 mg/kg & 3 mg/kg Q2W	100% receptor occupancy seen at 3 mg/kg	Anderson et al. SITC 2019 p260
Vibostolimab (MK-7684)	Phase I Multiple Solid tumors	2.1 mg to 700 mg Q3W RP2D: 200 mg Q3W	ORR 19% in combination with pembrolizumab Vibostolimab well tolerated as monotherapy and in combination with 200 mg pembrolizumab	Golan et al. SITC 2018
BMS-986207	Phase I/II	Not disclosed	No details	NCT02913313
EOS-448	Phase I	0.1 mg/kg, 1 mg/kg and 10 mg/kg	Receptor occupancy increased with dose. Nearly 100% occupancy was seen at 10 mg/kg dose. Dose-limiting toxicity was not seen.	Nguyen et al. AACR 2020
ASP-8374	Phase I Solid tumors	Not disclosed	Details not available	NCT03260322
COM-902	Phase I Solid tumors	7 doses to be tested for dose limiting toxicity. Q3W regimen	Data not available. Study posted in April 2020.	NCT04354246
Etigilimab	Phase I	0.3 mg/kg to 20 mg/kg Q2W	Safely administered up to 20 mg/kg. Stable disease was seen in 7/18 patients across all doses	Sharma et al. SITC 2018

**Table 5:** Ongoing clinical trials evaluating efficacy and safety of anti-TIGIT antibodies.

Drug; Sponsor	Clinical Trial Identifier; Phase	Study Title	Status as of August 2021
BMS-986207 Multiple Myeloma Research Consortium	NCT04150965; Phase I, II	Immuno-Oncology Drugs Elotuzumab, Anti-LAG-3, and Anti-TIGIT	Recruiting
BMS-986207; Compugen	NCT04570839 Phase I, II	COM701 in Combination With BMS-986207 and Nivolumab in Subjects With Advanced Solid Tumors.	Recruiting
IBI939; Innovent Biologics	NCT04353830; Phase I	A Study Evaluating the Safety, Tolerability, and Initial Efficacy of Recombinant Human Anti-T-cell Immunoreceptor With Ig and ITIM Domains (TIGIT) Monoclonal Antibody Injection (IBI939) in Subjects With Advanced Malignant Tumors	Recruiting
Ociperlimab; BeiGene	NCT04047862; Phase I	Study of BGB-A1217 in Combination With Tislelizumab in Advanced Solid Tumors	Recruiting
Ociperlimab; BeiGene	NCT04693234; Phase II	AdvanTIG-202: Anti-PD-1 Monoclonal Antibody Tislelizumab (BGB-A317) Combined With or Without Anti-TIGIT Monoclonal Antibody Ociperlimab (BGB-A1217) in Participants With Previously Treated Recurrent or Metastatic Cervical Cancer	Recruiting
Ociperlimab; BeiGene	NCT04732494; Phase II	AdvanTIG-203: Anti-PD-1 Monoclonal Antibody Tislelizumab (BGB-A317) Combined With or Without Anti-TIGIT Monoclonal Antibody Ociperlimab (BGB-A1217) in Participants With Recurrent or Metastatic Esophageal Squamous Cell Carcinoma	Recruiting
Ociperlimab; BeiGene	NCT04746924; Phase III	A Study of Ociperlimab With Tislelizumab Compared to Pembrolizumab in Participants With Untreated Lung Cancer	Recruiting
Ociperlimab; BeiGene	NCT04952597; Phase II	Study of Ociperlimab Plus Tislelizumab Plus Chemoradiotherapy in Participants With Untreated Limited-Stage Small Cell Lung Cancer	Recruiting
COM902; Compugen	NCT04354246; Phase I	COM902 (A TIGIT Inhibitor) in Subjects With Advanced Malignancies	Recruting
M6223; EMD Serono Research & Development Institute, Inc	NCT04457778; Phase I	First in Human Study of M6223 in Participants With Metastatic or Locally Advanced Solid Unresectable Tumors	Recruting
Tiragolumab; Genentech	NCT03563716 Phase II	A Study of MTIG7192A in Combination With Atezolizumab in Chemotherapy-Naïve Patients With Locally Advanced or Metastatic Non-Small Cell Lung Cancer	Active, Not Recruiting
Tiragolumab; Genentech	NCT04294810; Phase III	A Study of Tiragolumab in Combination With Atezolizumab Compared With Placebo in Combination With Atezolizumab in Patients With Previously Untreated Locally Advanced Unresectable or Metastatic PD-L1-Selected Non-Small Cell Lung Cancer (SKYSCRAPER-01)	Recruiting
Tiragolumab; Genentech	NCT04256421; Phase III	A Study of Atezolizumab Plus Carboplatin and Etoposide With or Without Tiragolumab in Patients With Untreated Extensive-Stage Small Cell Lung Cancer (SKYSCRAPER-02)	Recruiting
Tiragolumab; Hoffmann-La Roche	NCT03281369; Phase Ib, II	A Study of Multiple Immunotherapy-Based Treatment Combinations in Patients With Locally Advanced Unresectable or Metastatic Gastric or Gastroesophageal Junction Cancer (G/GEJ) or Esophageal Cancer (Morpheus-Gastric and Esophageal Cancer)	Recruiting
Tiragolumab; Hoffmann-La Roche	NCT04543617; Phase III	A Study of Atezolizumab With or Without Tiragolumab in Participants With Unresectable Esophageal Squamous Cell Carcinoma Whose Cancers Have Not Progressed Following Definitive Concurrent Chemoradiotherapy (SKYSCRAPER-07)	Recruiting
AB154; Arcus Biosciences	NCT03628677; Phase I	A Study to Evaluate the Safety and Tolerability of AB154 in Participants With Advanced Malignancies	Recruiting
AB154; Yale University	NCT04656535; Early Phase I	AB154 Combined With AB122 for Recurrent Glioblastoma	Recruiting
Vibostolimab; Merck	NCT02964013; Phase I	Study of Vibostolimab Alone and in Combination With Pembrolizumab in Advanced Solid Tumors (MK-7684-001)	Recruiting
Vibostolimab; Merck	NCT04165070; Phase II	Substudy 1: Efficacy and Safety Study of Pembrolizumab (MK-3475) Plus Chemotherapy When Used With Investigational Agents in Treatment-Naïve Participants With Advanced NonSsmall Cell Lung Cancer (NSCLC) (MK-3475-01A/KEYNOTE-01A)	Recruiting
Vibostolimab; Merck	NCT04305041; Phase I, II	Substudy 02A: Safety and Efficacy of Pembrolizumab in Combination With Investigational Agents in Participants With Programmed Cell-Death 1 (PD-1) Refractory Melanoma (MK-3475-02A)	Recruiting
Vibostolimab; Merck	NCT04305054; Phase II	Substudy 02B: Safety and Efficacy of Pembrolizumab in Combination With Investigational Agents or Pembrolizumab Alone in Participants With First-Line (1L) Advanced Melanoma (MK-3475-02B)	Recruiting
Vibostolimab; Merck	NCT04303169; Phase II	Substudy 02C: Safety and Efficacy of Pembrolizumab in Combination With Investigational Agents or Pembrolizumab Alone in Participants With Stage III Melanoma Who Are Candidates for Neoadjuvant Therapy (MK-3475-02C)	Recruiting
ASP8374; Astellas Pharma	NCT03260322; Phase I	A Multiple-dose Study of ASP8374, an Immune Checkpoint Inhibitor, as a Single Agent and in Combination With Pembrolizumab in Subjects With Advanced Solid Tumors	Recruiting

## Data Availability

All the data generated during the review are included in the illustrations.
